# Neutrons describe ectoine effects on water H-bonding and hydration around a soluble protein and a cell membrane

**DOI:** 10.1038/srep31434

**Published:** 2016-08-16

**Authors:** Giuseppe Zaccai, Irina Bagyan, Jérôme Combet, Gabriel J. Cuello, Bruno Demé, Yann Fichou, François-Xavier Gallat, Victor M. Galvan Josa, Susanne von Gronau, Michael Haertlein, Anne Martel, Martine Moulin, Markus Neumann, Martin Weik, Dieter Oesterhelt

**Affiliations:** 1 Institut Laue Langevin, F-38042 Grenoble, France; 2 CNRS, IBS, F-38044 Grenoble, France; 3 CEA, IBS, F-38044 Grenoble, France; 4 Univ. Grenoble Alpes, IBS, F-38044 Grenoble, France; 5 Bitop, Stockumer Str. 28, 58453 Witten, Germany; 6 Institut Charles Sadron CNRS–UdS, 23 rue du Loess, BP 84047, 67034 Strasbourg Cedex 2, France; 7 Max-Planck-Institute of Biochemistry, Department of Membrane Biochemistry, Martinsried, Germany

## Abstract

Understanding adaptation to extreme environments remains a challenge of high biotechnological potential for fundamental molecular biology. The cytosol of many microorganisms, isolated from saline environments, reversibly accumulates molar concentrations of the osmolyte ectoine to counterbalance fluctuating external salt concentrations. Although they have been studied extensively by thermodynamic and spectroscopic methods, direct experimental structural data have, so far, been lacking on ectoine-water-protein interactions. In this paper, *in vivo* deuterium labeling, small angle neutron scattering, neutron membrane diffraction and inelastic scattering are combined with neutron liquids diffraction to characterize the extreme ectoine-containing solvent and its effects on purple membrane of *H. salinarum* and *E. coli* maltose binding protein. The data reveal that ectoine is excluded from the hydration layer at the membrane surface and does not affect membrane molecular dynamics, and prove a previous hypothesis that ectoine is excluded from a monolayer of dense hydration water around the soluble protein. Neutron liquids diffraction to atomic resolution shows how ectoine enhances the remarkable properties of H-bonds in water—properties that are essential for the proper organization, stabilization and dynamics of biological structures.

Halophilic eubacteria isolated from salt marshes or marine environments include a variety of interesting species of high biotechnological concern such as the recently discovered rust-producing *Halomonas titanicae* in the hull of the Titanic^1^. In order to compensate for fluctuations in the osmolarity of the medium, their cytosol can produce ectoine, up to an intracellular concentration of 20% of the cellular dry mass^2^,^3^,^4^. By this adaptive regulatory process the microorganism is said to be *halotolerant* over a broad range of salt concentration (*e.g.* 0.5 to 25% NaCl). Ectoine is called a *compatible solut*e in the sense that its occurrence at molar concentrations in the cytoplasm does not interfere with cellular biochemistry and metabolism^5^,^6^,^7^. Compatible solutes, in general, constitute a set of low molecular weight molecules belonging to different chemical families, such as polyoles, sugars, amino acids, betaines and ectoines, which maintain osmotic equilibrium in many halophilic and dry-tolerant bacteria, algae and fungi. Their effects on proteins have been studied in detail mainly by thermodynamic and spectroscopic methods^5^,^6^,^7^,^8^,^9^. The current working concept is that (i) compatible solutes strengthen native protein structures by promoting a more compact conformation and (ii) solute-water ‘binding’ leads to preferential hydration of the proteins according to the “preferential exclusion” model of Arakawa and Timasheff^10^. The model, in which the solute is preferentially excluded from the hydration shell around the protein surface, is comforted by vapor pressure osmometry results showing negative preferential interaction coefficients between solute and protein^11^,^12^ and 1 ns molecular dynamics simulations^13^. An early small angle neutron scattering (SANS) experiment provided experimental evidence for such a structure around ribonuclease in aqueous solvent containing glycerol^14^. And hydration shells around halophilic proteins^15^, and mesophilic proteins^16^ have been characterized by a combination of small angle neutron and X-ray scattering.

Ectoine, which is a widely occurring osmolyte, has a very high solubility (~4 mol/L) in water at 20 °C. Similarly to other compatible solutes, it has a stabilizing effect on proteins and membranes^5^,^17^,^18^ and a related inhibitory effect on inflammation in mammalian cells caused by external stress factors has been demonstrated^19^. Biocompatibility tests have characterized ectoine as a virtually physiologically inert compound up to high concentrations. The molecule is not only a prominent and widely distributed compatible solute but also a substance that through its hydration, stabilization and anti-inflammatory properties has found broad cosmetic and clinical applications^18^,^20^,^21^,^22^. Consequently, the genomics, biochemistry and biotechnological production of ectoine from its main producer, *Halomonas elongate*, have been studied to great detail (reviewed in refs 7, 23 and 24). Spectroscopic experiments and MD simulations^8^,^9^,^25^,^26^,^27^ suggested a pronounced ordering of H-bonds in a well-defined hydration sphere around the ectoine molecule and its preferential exclusion from protein surfaces. In the model, the large water/ectoine clusters formed would not fit the surfaces of proteins and membranes^8^. In a recent paper, Hahn *et al*.^9^ combined surface plasmon resonance, confocal Raman spectroscopy, molecular dynamics simulations, and density functional theory calculations to study the local hydration shell around ectoine and its influence on the binding of a gene-5-protein to a single-stranded DNA. So far, however, direct experimental structural data on ectoine hydration and its interactions with macromolecules have been lacking.

Neutron scattering and diffraction are powerful methods for the characterization of structure and dynamics of biological molecules^28^,^29^,^30^, liquids^31^ and bound water^32^,^33^,^34^. Neutron wavelengths are in the ~1–10 Å range corresponding to the atomic and molecular length scale, while neutron energies correspond to the picosecond to nanosecond time scale of molecular dynamics (MD). Neutrons are scattered by atomic nuclei with isotopes of the same element having different scattering amplitudes. Hydrogen and deuterium, in particular, are clearly distinguishable and H/^2^H(D) labeling greatly enriches the information obtained by neutron scattering on complex systems.

Within the assumption that, apart from molecular crowding effects found in all cells, the properties of the halotolerant aqueous intracellular environment is dominated by the presence of ectoine, the aim of the present work is to provide a structural characterization of molar ectoine aqueous solvents as well as of their effects on a soluble protein and a membrane. *E.coli* maltose binding protein (MBP) (calculated pI 5.47)), which can be obtained with various levels of deuterium labeling, has been used extensively as a model for biophysical studies^35^, while the hydration dependence of structure and dynamics of purple membranes of *Halobacterium salinarum* (PM) is currently the best characterized for a natural membrane^36^,^37^,^38^.

(i) The hydration shell around MBP in solution with ectoine was measured by SANS, by using natural abundance and deuterated protein and H_2_O/D_2_O contrast variation^29^;

(ii) PM occur naturally as highly ordered two-dimensional crystalline patches of bacteriorhodopsin and lipids. The location of ectoine in the direction normal to the surface of PM as well as on the membrane plane was determined by neutron membrane diffraction, by using natural abundance and deuterium labeled ectoine and H_2_O/D_2_O exchange;

(iii) The picosecond to nanosecond dynamics of PM in the presence of ectoine was measured by energy resolved incoherent neutron scattering^28^;

(iv) Finally, neutron liquids diffraction^39^ was used to examine how water structure is modified in molar solutions of ectoines to interatomic resolution, again by using deuterium labeled ectoine and H_2_O/D_2_O exchange.

The results provide model-independent, quantitative, structural evidence for preferential exclusion of ectoine from soluble protein and membrane surfaces. Furthermore, the effect of ectoine on water H-bonding, characterized by the liquids diffraction experiment, suggests an explanation for why ectoine constitutes an appropriate compatible osmolyte for the cytosolic halotolerant response to high extracellular salt concentrations.

## Results and Discussion

### Hydration of a soluble protein in the presence of ectoine seen by SANS

**Figure 1:**
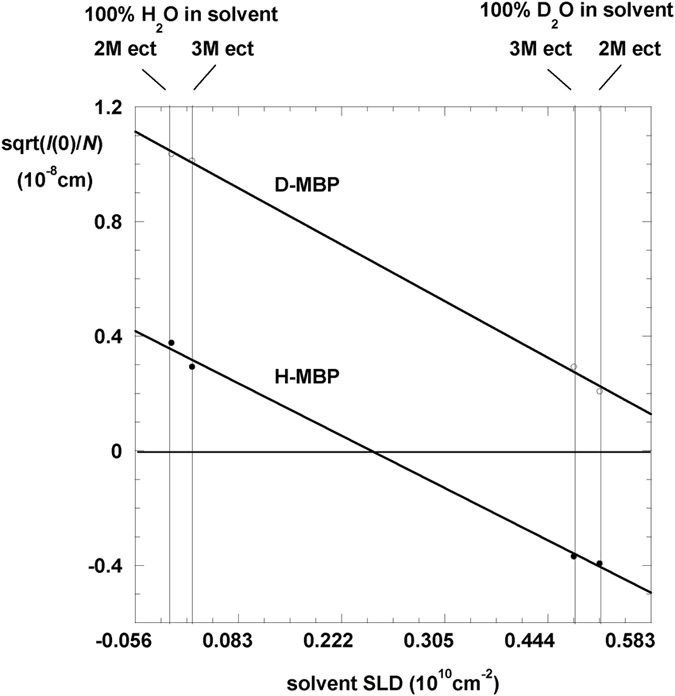
Stuhrmann plots H- and D-MBP in various solvents. Square root of corrected forward scattered intensity (equal to particle excess scattering length Δ*ρ*V) *versus* solvent SLD for H-MBP and D-MBP in 2 M ectoine and 3 M ectoine H_2_O and D_2_O solvent.

MBP is a soluble protein of 387 amino acids and molecular mass 42490 Da^38^. For a macromolecule in solution, *contrast* is defined as the difference between its scattering length density (SLD) and that of the solvent (see Methods). By applying the Guinier approximation (see Methods), SANS from a dilute monodisperse, macromolecular solution provides two model-independent experimental parameters on an absolute scale: the forward scattered intensity *I*(0) (cm^−1^), related to the concentration, molecular volume and SLD contrast of the particle in solution, and the square of the radius of gyration *R*_g_^2^ (Å^2^) of contrast within the particle. Note that ‘particle’ refers not to the protein alone but to the volume of SLD different from that of bulk solvent, *i.e.* the macromolecule plus its hydration shell if that is different from bulk solvent. Four solvent conditions were examined for H-MBP and D-MBP on the D22 camera at the Institut Laue Langevin in Grenoble (ILL): 2 M and 3 M H-ectoine in 100% H_2_O and 100% D_2_O. The addition of ectoine led to significantly different solvent SLD values (Table 1), providing 4 contrast data points for each of H-MBP and D-MBP. The measured parameters on an absolute scale are in Table 1 with the straight-line Stuhrmann plot (see Methods) in Fig. 1.

**Figure 2:**
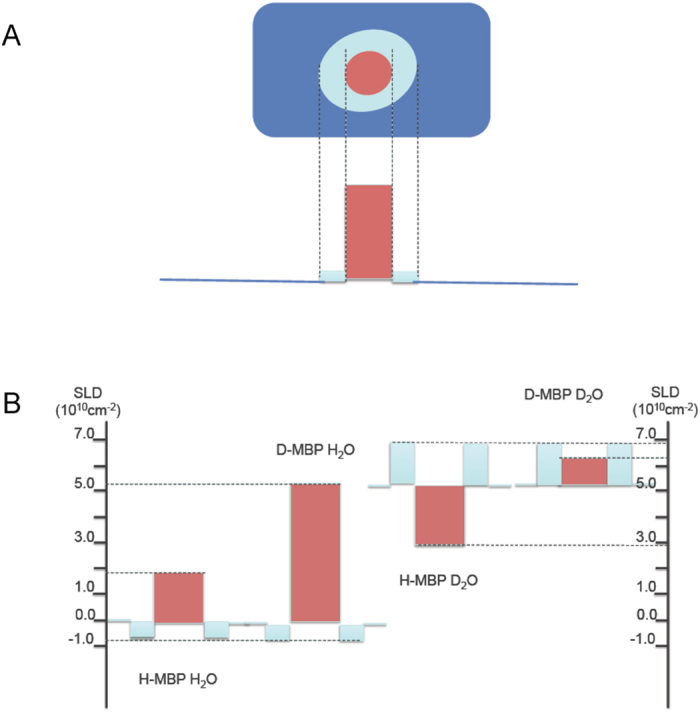
(**A**) Schematic diagram of the particle made up of protein (red) surrounded by its hydration shell (light blue); the solvent is shown as dark blue. (**B**) Section showing the SLD distribution through solvent and H-MBP and D-MBP particles in 3 M ectoine in H_2_O and D_2_O. The particles are divided into their protein (red) and hydration shell of specific gravity 1.1 (blue) components. Solvent SLD levels are shown as straight lines at 0 and 5.456 × 10^10^ cm^−2^.

The measured values were put on an absolute scale following the method of Jacrot and Zaccai^40^, and were interpreted in terms of the SLD distribution in a MBP particle composed of the protein component surrounded by a hydration shell (Fig. 2A). A SLD distribution with no hydration shell around the protein does not fit the observed intensity or radius of gyration values as a function of contrast for the different samples. Details of the fitting procedure are given in Methods. The SLD distribution illustrated in Fig. 2B provides the best quantitative fit to the data. A dense water hydration shell^16^, of volume (0.30 ± 0.05 x the protein volume) and of radius of gyration 33 ± 1 Å surrounds the protein of radius of gyration 25 ± 1 Å. This corresponds to exclusion of the molar ectoine solvent by about one molecular layer (about 3 Å thick) of dense water around the protein surface.

### Neutron membrane diffraction shows ectoine is excluded from the surface of purple membranes (PM)

**Figure 3:**
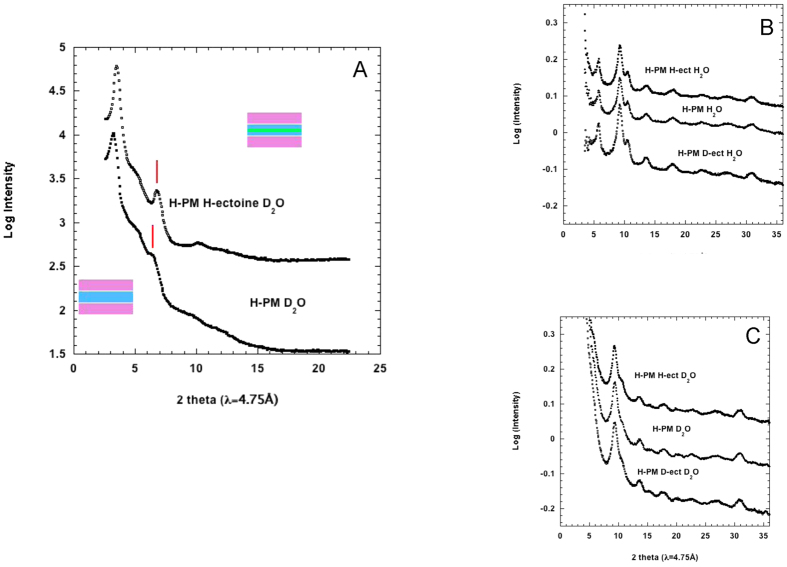
(**A**) Lamellar diffraction from PM oriented membrane stacks under different conditions plotted against scattering angle (2*θ* in degrees). The main lamellar peaks correspond to a spacing of d ~ 82 Å. Note the significant increase in second order (indicated by red lines) intensity when H-ectoine is added to D_2_O in the inter-lamellar hydration layer. The schematic diagram shows two membranes (purple) in the stack separated by a water layer (light blue); the green line in the top diagram indicates the position of ectoine. (**B**) In-plane diffraction for samples hydrated with H_2_O, and (**C**) for samples hydrated with D_2_O.

The D16 diffractometer at the ILL was originally developed to study PM structure and hydration^41^. Neutron diffraction from specifically labeled PM on the membrane-diffractometer has provided the location of various membrane components *perpendicular to* as well as *in the membrane plane* structure (e.g. see refs 36, 42 and 43). Diffraction patterns from stacks of H-PM hydrated in H_2_O or D_2_O, and by 1 M D- or H-ectoine in H_2_O or D_2_O are shown in Fig. 3. The position of ectoine in the hydration layer between membranes was obtained from the lamellar analysis of the D_2_O samples (where the contrast of H-PM and H-ectoine is highest). Compared to PM in absence of ectoine, the sample including ectoine has a slightly smaller lamellar periodicity and is better ordered with sharper peaks. The more pronounced second order (red lines in Fig. 3A) is reminiscent of neutron diffraction from myelin membranes, in which a stronger second order in D_2_O compared to H_2_O showed that the water layer was located at half the unit cell^44^. The data in Fig. 3A indicate a predominant positioning of the H-ectoine in the middle of the solvent layer at half the lamellar periodicity (the green line in the membrane diagram). Weak shoulders at scattering angles 5° and 10° show the existence of a minor fraction of weakly hydrated membrane stacks with d ~ 54 Å (the thickness of the ‘dry’ membrane is ~50 Å). In-plane diffraction from PM is dominated by contrast between the protein and lipid areas in the projection. The similarity of diffraction from the six samples, H-PM with H-ectoine or D-ectoine, and in absence of ectoine, in H_2_O (Fig. 3B) and D_2_O (Fig. 3C) indicates that the two-dimensional structure is fully preserved and there is no change in contrast between the protein and lipid areas, *i.e.* no preferential binding of ectoine either on the protein or on the lipid areas of the purple membrane surface.

### The presence of ectoine does not influence the dynamics of PM

**Figure 4:**
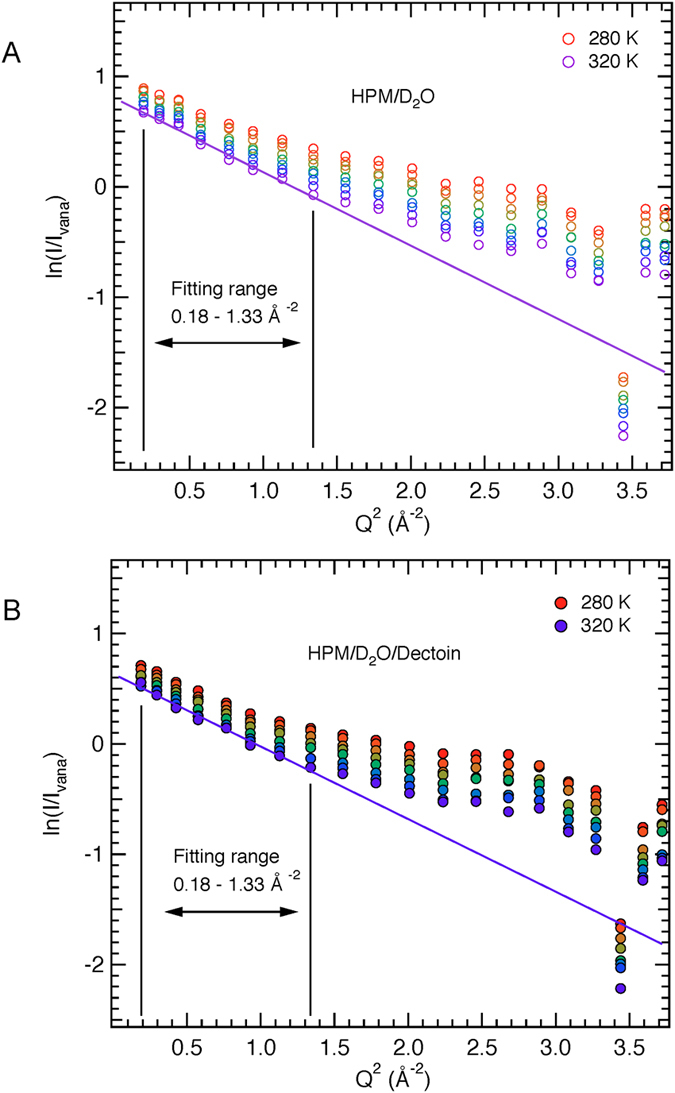
Logarithmic representation of the normalized intensities as a function of *Q*^2^ at eight temperatures between 280 and 320 K for (**A**) H-PM/D_2_O and (**B**) H-PM/D_2_O/D-ectoine (*b*). The *Q*^2^ range from which the MSD were extracted (0.18 Å^−2^ < Q^2^ < 1.33 Å^−2^), according to the Gaussian approximation (equation 9) is indicated by vertical lines. The straight lines in (**A**,**B**) indicate the linear fits at 320 K.

Neutrons give up or gain energy and momentum when they collide with moving atoms. The IN16 spectrometer at ILL allows measurements of the scattered intensity with very good energy and momentum resolution to provide information in the nanosecond time-scale on sample molecular dynamics. PM dynamics as a function of hydration has been studied extensively by the incoherent neutron scattering method (see refs 37, 38 and 45). The incoherent scattering cross-section of H is more than an order of magnitude larger than for other nuclei in the sample including D, and deuterated ectoine and D_2_O were used in order to reduce the contribution of ectoine and water dynamics. The data permitted to focus, therefore, on the motions of natural abundance H in the membrane lipids and protein. The scattered intensity measured as a function of temperature, for D_2_O-hydrated PM in absence of ectoine and in the presence of D-ectoine, is plotted on a log scale as a function of scattering vector (Q) squared, in Fig. 4. Clearly, the data are closely alike for the two samples, indicating essentially identical dynamics on the nanosecond time-scale. Ectoine, which diffraction experiments showed to be excluded from the membrane surface hydration, therefore, does not change the dynamics of PM on the nanosecond time-scale.

**Figure 5:**
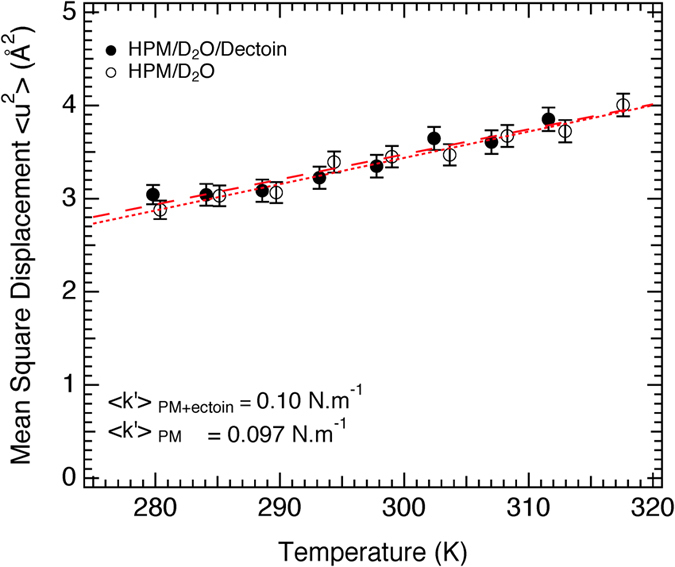
Mean square displacements as a function of temperature (from the data in Fig. 4) and calculated resilience values (<k′>) on the nanosecond time scale for H-PM samples hydrated with D_2_O in the presence and absence of D-ectoine (see text).

Mean square displacements (MSD in Å^2^ units) and an effective molecular resilience (<*k*′> in N/m units) can be obtained from the temperature dependence of elastic incoherent scattering^46^. MSD and <*k*′> values for the two samples, calculated from the Gaussian approximation to the low *Q* range (see Methods), are shown in Fig. 5. They are similar to values found previously of hydrated PM samples and quoted in ref. 46.

### The presence of ectoine favors weaker water-water intermolecular H-bonds

**Figure 6:**
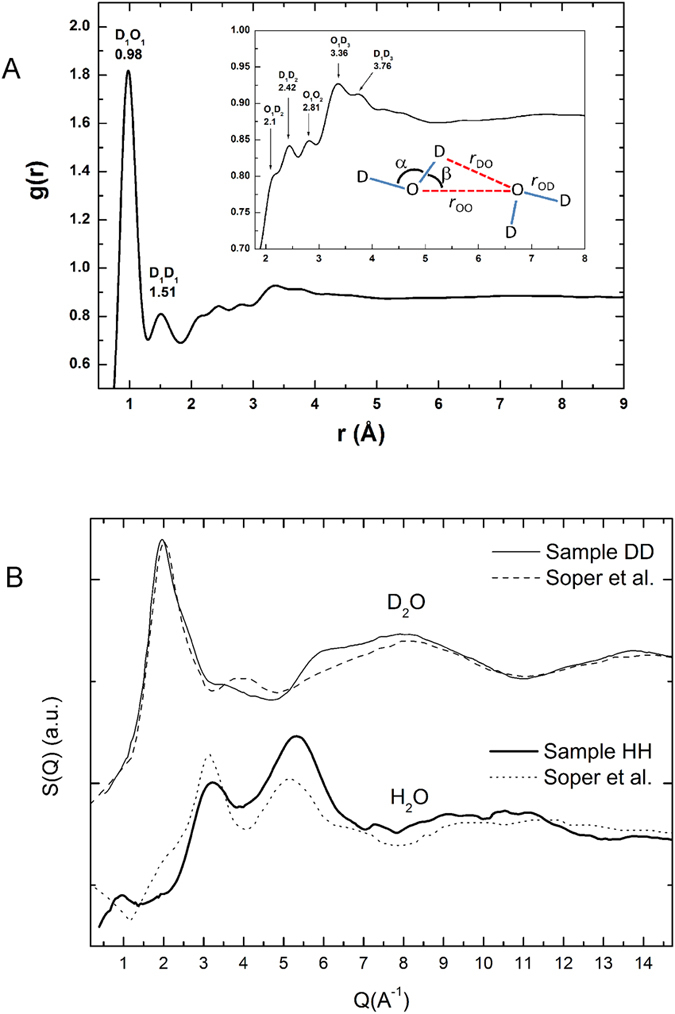
(**A**) Liquids diffraction of ectoine solutions. The radial distribution function g(r), obtained by Fourier transformation of the experimental structure factor S(Q) for sample 1.5 M D-ectoine/D_2_O. The inter-molecular region, beyond r = 2 Å, is expanded in the inset, which also shows the H-bonding scheme between two adjacent D_2_O molecules (see text). (**B**) Comparison between the structure factor of the D-labeled ectoine in D_2_O (labeled DD) solution and the corresponding pure D_2_O from Soper *et al*.^48^ (upper curve), and D-labeled ectoine in H_2_O (labeled HH) and the corresponding pure H_2_O (bottom curve). Significant differences between the ectoine solutions and pure water are apparent.

In order to explore the effect of molar concentrations of ectoine on water structure, aqueous solutions of 1.5 M ectoine were examined on the D4 liquids diffractometer (see Methods): natural abundance ectoine in heavy water (D_2_O) (H-ectoine/D_2_O), and D-labeled ectoine in D_2_O (D-ectoine/D_2_O). The aim of the experiment was to analyze structure modifications of H-bonding in water through the radial distribution functions, g(r), obtained by Fourier transformation of the experimental structure factors S(Q). Data were collected also from the corresponding natural abundance (H-ectoine/H_2_O) sample, but were too noisy to be analyzed reliably, because of high incoherent scattering background (Supplementary Figure S1). The g(r) for sample D-ectoine/D_2_O is in Fig. 6A. Features in the g(r) between 2 and 4 Å (Fig. 6A) correspond to correlations in this distance range. Intra-molecular correlations within the ectoine molecule have been calculated and subtracted by the method of Talón *et al*.^47^. In order to assess ectoine-ectoine intermolecular correlations, we have estimated the average distance between solute molecules from the solution concentration. At 1.5 moles-per-liter, the solution consists of 31 moles of water per mole of ectoine. Hahn *et al*.^9^ have estimated 8 water molecules in direct contact with one molecule of solute, most of which are also associated with each other so we expect about 16 water molecules in a second shell, assuming tetrahedral coordination. The remaining 7 water molecules lie beyond. In any given direction, there will be, on average, >2 shells of water, around each ectoine molecule, and >4 between neighboring ectoine molecules so that ectoine-ectoine intermolecular contributions would appear at further than 4 Å in the g(r), beyond the range analyzed in Fig. 6A. To a good approximation, therefore, (after subtraction of ectoine intra-molecular correlations) the radial distribution function in Fig. 6A is dominated by correlations between neighboring heavy water molecules in the two successive shells around the solute.

We could assign the main peaks in the g(r) by comparing directly with partial distribution functions experimentally obtained by Soper *et al*. (Fig. 6B)^48^. In Table 2, the parameters obtained from the analysis of ectoine solutions are compared to *average* parameters for an instantaneous H-bond configuration in liquid water calculated by Modig *et al*.^49^. Intra-molecular contributions from OD (0.98 Å) and DD (1.51 Å) bonds with a D-O-D angle of 100.4° are apparent in the range r < 2 Å in the DD g(r). In the intermolecular r~2–4 Å range, the peaks associated with O-O bonds (2.81 Å) (in the D-ectoine/D_2_O g(r)) and O-D2 bonds (1.93 Å) (in the HD g(r)) provide information on the average ordering between two adjacent water molecules in the solution. In particular, the measured distances result in a H-bond angle of 148° (or 22.36° if it is considered as the angle between the O-H and the O-O vectors) (Fig. 6A inset). The experimental structure factor (and thus the corresponding radial distribution function) contains information about the average correlation distances from the water between ectoine molecules.

Liquid water consists of a dynamic mixture of strong, short, straight and weaker long, bent H-bonds^50^. Recall that it is the *average* parameters for an instantaneous H-bond configuration in the liquid that are given in Table 2 and compared to H-bonding in water (see for example, http://www1.lsbu.ac.uk/water/water_hydrogen_bonding.html). The larger beta angle will move the H atom away from the O atom in the neighboring molecule (inset in Fig. 6A), decreasing the screening of the negative repulsion between O atoms. As a consequence, these water molecules move away from each other, leading to a longer O-D2 distance (Table 2), an effect that is interpreted as a weakening of the H-bond. The mean geometrical distances and angles obtained by neutron diffraction for the ectoine solutions established a significantly more bent *average* water-water intermolecular H-bond configuration than in bulk water, *i.e.* a larger proportion of weaker H-bonds in presence of ectoine favor greater configuration exchange in the water H-bond structure thus contributing entropy to the thermodynamic preference for ectoine to be excluded from the surface of proteins and membranes. Previous IR^8^,^9^ and MD^25^,^26^ results showed strong H-bonding of water to ectoine. A combination of the neutron, IR and MD results favors a model in which the modification of water structure by ectoine appears to be similar to that of water confined in reverse micelles, in which were observed strong water-polar group H-bonds as well as a larger proportion of inter-water H-bonds with energetically unfavorable angles compared to bulk water^51^.

Important contributions to the large solubility of ectoine and its preferential exclusion from the surfaces of MBP and purple membranes then appear through two distinct H-bond effects: (i) Favorable enthalpy change from ordering of water molecules though stronger water-ectoine H-bonds; (ii) Favorable entropy through the weakening of water-water H-bonding in the vicinity of the solute.

## Conclusion

SANS and membrane diffraction data provide model-independent, quantitative, structural evidence for preferential exclusion of ectoine from the surfaces of MBP and PM, respectively. Energy-resolved neutron scattering data show that membrane molecular dynamics is not affected by the presence of ectoine in the solvent environment. The liquids diffraction data on molar ectoine aqueous solution establish a significant weakening of inter-water H-bonding in the vicinity of ectoine. The combination of neutron with IR^8^,^9^ and MD^25^,^26^ results suggests a picture in which the large solubility of ectoine and its preferential exclusion from the hydration shell of MBP and purple membranes are driven by *both* H-bond ordering from direct interaction with ectoine (a favorable enthalpy term) *and* through the weakening of water-water H-bonding (a favorable entropy term).

A motivation behind this study was the assumption that the properties of the molar ectoine aqueous environment contribute significantly to solvent properties of the cytoplasm in *Halomonas*. The evidence that in mesophile cells cytoplasmic water (*in vivo*) is essentially identical to bulk water (*in vitro*) supports this assumption. The dynamics of cytoplasmic water has been studied *in cellula* by neutron scattering in red blood cells^52^ and by neutron scattering^53^,^54^ and NMR^55^ in *E. coli*. In all three studies, it was found that about 90% of intracellular water behaves as bulk water with only the remaining 10% corresponding to interfacial water that can be accounted for by macromolecular hydration layers. In fact, bulk water has remarkable properties, based on dynamic H-bond networks that play vital roles in macromolecular folding and interactions^56^. As the name indicates ‘compatible’ solutes do not interfere with the essential properties of bulk and hydration water but preserve and may even reinforce them. H-bond thermodynamics, for example, is an important component of the hydrophobic effect^56^, while water rotational diffusion in hydration shells contributes entropy to drive functional protein dynamics^34^. The results presented in this paper illustrate how the osmolyte behind the halotolerance response in microorganisms induces compensating effects on water H-bonding that respect its remarkable properties, without penetrating into macromolecular hydration shells.

The combination of neutron methods presented in the study paves the way for similar studies of solvent environment effects on protein and membrane interactions. For example, in the context of the mainly acidic nature predicted for 3474 putative proteins (main peak at pI 4.7, median pI 6.32) in the *H. elongata* proteome^57^, we recall that MBP (pI 5.47) is also acidic while PM is from an extreme halophile. Unlike extreme halophilic archaea, however, the cytosol of *Halomonas* does not contain molar KCl, since the main osmolyte is ectoine. It would, nevertheless, be of considerable interest to repeat all the experiments in this work in the presence of ectoine *and* various concentrations of KCl and NaCl, respectively. Further to their participation in electrostatic interactions with charged proteins and nucleic acids, Na^+^ and K^+^ are known to affect water H-bonding differently (e.g. see ref. 15).

## Methods

### Deuterated ectoine from Halomonas

As soon as the C-source was used up, the cells were harvested through centrifugation. D-ectoine was extracted from the cell pellet through osmotic downshock (re-suspension of the cells in deionized water). The extract was acidified (to pH 1.6) and filtered through paper a filter to remove the protein precipitate. The D-ectoine was purified through capture on cation-exchange resin Dowex Marathon C (Na^+^), elution with 0.2 N NaOH, drying and re-crystallization from methanol. The resulting purity was 91% (w/w)^58^.

### Natural abundance and deuterium labeled Maltose-Binding Protein

Natural abundance (H-MBP) and 100% D_2_O ‘scattering length density (SLD) contrast match-out’ (see SANS section below) labeled Maltose Binding Protein (D-MBP) were expressed in the Deuteration Laboratory of the Institut Laue Langevin (ILL) in a kanamycin selected High Cell Density culture as described in ref. 38. For the deuterated culture, natural abundance glycerol was used as carbon source and the D_2_O content of the growth medium was fixed at a level of 85% to allow matching-out of D-MBP in 100% D_2_O.

### Purple membrane of Halobacterium salinarum

Natural abundance *H. salinarum* were cultured and purple membranes (PM) extracted as described previously^59^. Samples for membrane diffraction and inelastic scattering on the D16 diffractometer and IN16 back-scattering spectrometer at ILL, respectively, were prepared follows: 150 mg of PM was solubilized in H_2_O or D_2_O before being pelleted by centrifuging at 20,000 RPM for 1 hour. This procedure was repeated 3 times in order to ensure a complete H/D exchange of the protein exchangeable hydrogen atoms. Natural abundance (H-) or D-ectoine was then added to the PM solution in such quantity that the concentration of ectoine in the final sample would be 1 M. The mixture was then spread in a 4 × 3 cm^2^ flat aluminum sample holder, before being dried progressively over P_2_O_5_ until it reached the hydration level of 2 g of 1 M ectoine/water per gram of PM. The sample holder was finally sealed by an aluminum cover (0.3 mm neutron path length) using indium wire of 1 mm diameter.

### Small Angle Neutron Scattering

Experiments were performed at the ILL on the D22 SANS camera (http://www.ill.eu/d22). *Scattering length density* (SLD; note that for X-rays, SLD is proportional to electron density) is an important concept for the interpretation of neutron or X-ray small angle scattering experiments on particles in solution. The intensity as a function of scattering vector modulus (*Q* = 4 π sin θ/λ, 2θ is scattering angle and λ is radiation wavelength) contains information on the distribution of SLD *contrast* in the particle, where SLD contrast is equal to the difference between particle and solvent SLD. In the method of *contrast variation*, scattering from volumes of different SLD within a particle can be enhanced or diminished by appropriately adjusting the SLD of the solvent. For X-rays, for example, this can be done by adding solutes of different electron density; for neutrons, by adjusting the H_2_O/D_2_O ratio, since the neutron scattering lengths of H and D are significantly different^29^.

Data were put on an absolute scale following Jacrot and Zaccai^40^ and analyzed in the Guinier approximation^60^


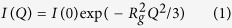


to yield the forward scattered intensity, *I*(0), and, *R*_g_, radius of gyration of SLD contrast, for each sample condition. *I*(0) were subsequently analyzed by contrast variation using Stuhrmann plots^29^:


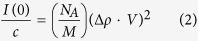



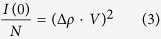


where *c* (g/cm^3^) is protein concentration, *N*_*A*_/*M* is Avogadro’s number/protein molar mass (g^−1^), this term converts *c* into *N,* number concentration in equation (3)*ρ* (cm^−2^) is SLD contrast and *V* (cm^3^) is particle volume. In a Stuhrmann plot, the square root of *I*(0)/*N* is plotted against solvent SLD to yield a straight line of slope depending on particle *V* and labile H exchange (Fig. 1); solvent SLD at Δ*ρ* = 0 is called the ‘contrast match’ point of the particle, where its average SLD equals that of the solvent.

The particle consists of the protein and its hydration shell, not of the protein alone, so that for each contrast condition, we can write


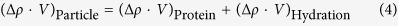


Samples were in either 100% H_2_O or 100% D_2_O, and contrast was varied by the addition of either 2 M or 3 M ectoine to the solvent, to give four data points each for H-MBP and D-MBP, respectively (Fig. 1). Contrast variation plots in absence of ectoine in H_2_O/D_2_O mixed solvents were also measured for H-MBP and D-MBP, indicating match points of 40% and 100% D_2_O, respectively (not shown). These values are in accordance with calculated values of (Δ*ρ* · *V*)_Protein_ of H-MBP and D-MBP, from chemical composition, a partial specific volume of 0.75 cm^3^g^−1^, and 85% exchange of labile H atoms in the protein^40^. In 100%H_2_O, (Δ*ρ* · *V*)_Particle_ values are less sensitive to particle volume or hydration effects. For the data in ectoine containing solvents, it was first checked that the observed (Δ*ρ* · *V*)_Particle_ values, calculated from equation (2) for H-MBP and D-MBP in 100%H_2_O, were close to calculated values for (Δ*ρ* · *V*)_Protein_. The control confirmed that the solution was well behaved for Guinier analysis (monodisperse MBP monomers with no inter-particle interaction).

The analysis was pursued to fit hydration shell parameters by re-writing the hydration term in equation (4):


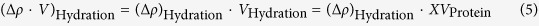


In the assumption of a pure water hydration shell, (Δ*ρ*)_Hydration_ is the difference in SLD between 100% H_2_O or 100% D_2_O and ectoine containing solvent. A combination of small angle X-ray and SANS experiments had shown that protein hydration water was denser by about 10% than bulk water^16^ and in the following fitting procedure (Δ*ρ*)_Hydration_ was adjusted accordingly.

*X*, the ratio (*V*_Hydration_/*V*_Protein_), is the parameter to be fitted; *X* = 0 would correspond to no hydration shell.

Equation (6) was used to find the values of *X* that best fit the experimental observations.


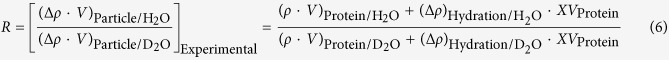


**Figure 7:**
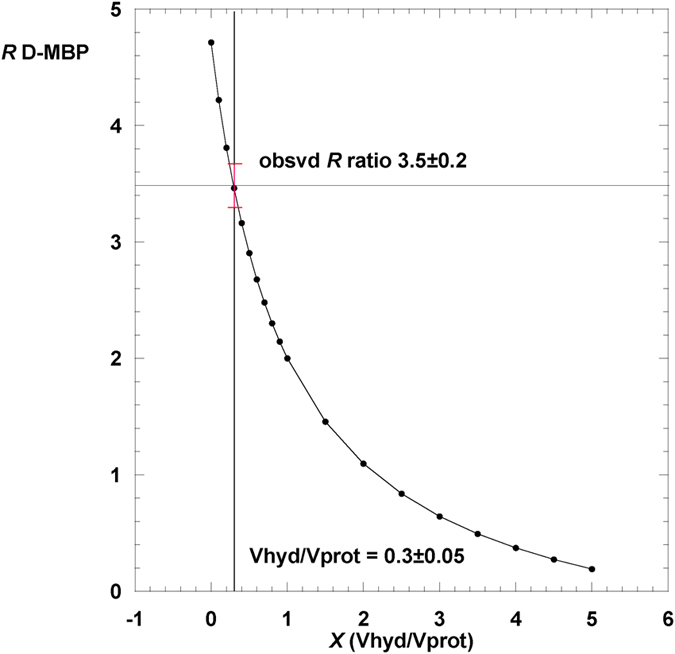
Plot of the ratio (Δ*ρ*V in 3 M ectoine H_2_O)/(Δ*ρ*V in 3 M ectoine D_2_O) *vs X (V*_Hydration_/*V*_Protein_) for D-MBP. The red point with error bars indicates the experimental value of the ratio and corresponding *X* value. See Methods text.

The experimental ratio *R* on the left hand side of the equation was taken from Fig. 1 for each sample and contrast condition. All the terms on the right hand side (except for *X*) were calculated from the chemical compositions and partial specific volumes of protein and solvent components. For each of H-MBP and D-MBP, a graph of *R vs X* was plotted, to derive the values of *X* that agreed best with the observed *R* and its error bar. D-MBP data were the most discriminating. The plot for D-MBP in 3 M ectoine is in Fig. 7, showing that the best fit is for *X* = 0.30 ± 0.05.

Since *X* in equation (6) multiplies the product (Δ*ρ* · *V*) it could just as well imply, for example, the existence of a hydration shell of smaller (Δ*ρ*) and larger volume. In such a case, the hydration shell would extend further and have a larger radius of gyration. In order to distinguish between scenarios, the radius of hydration of the hydration shell was calculated from the parallel axes theorem analysis of radii of gyration as a function of contrast^61^:


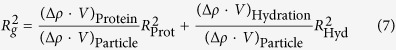


Equation (7) is valid in the reasonable assumption that centers of mass of protein and hydration shell coincide.

Recall that the particle term is the sum of the protein and hydration terms, so that the equation can be re-written:


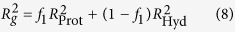


where *f*_1_ is the fractional protein contribution at each contrast condition.

A straight line fit to the observed *R*_*g*_^2^ (Table 1) *vs f*_1_ yielded

*R*_Prot_^2^ 25 ± 1 Å (extrapolated at *f*_1_ = 1) and

*R*_Hyd_^2^ 33 ± 1 Å (extrapolated at *f*_1_ = 0)

These values are fully consistent with a hydration shell made up of monolayer water (3 Å thick) surrounding the protein.

### Membrane neutron diffraction

Neutron diffraction experiments on oriented PM samples were carried out on the D16 diffractometer at ILL (http://www.ill.eu/d16). The wavelength was 4.75 Å (Δλ/λ = 0.01) and the scattering geometry was set with the neutron beam focused vertically to the sample and slit collimated in the horizontal direction. Diffraction patterns were collected using the Millimeter Resolution Large Area Neutron Detector (MILAND), a high pressure ^3^He neutron detector with an area of 320 mm × 320 mm and a “pixel” resolution of 1 mm × 1 mm. The sample-to-detector distance was 910 mm. Data analysis was performed with standard D16 software. Lamellar peaks, corresponding to one dimensional crystalline order perpendicular to the plane of the membrane, and in-plane diffraction peaks, corresponding to two-dimensional crystalline order in the membrane plane, were analyzed by calculating the corresponding crystal spacings from Bragg’s law [Chapter G1 in refs 41 and 61].

### Neutron spectroscopy

Elastic incoherent neutron scattering experiments on D_2_O hydrated natural abundance PM in the presence or absence of D-ectoine (H-PM/D_2_O, H-PM/D_2_O/D-ectoine, respectively) were carried out on the backscattering IN16 spectrometer^62^. Each sample was inserted at room temperature into an Orange cryostat at 135° with respect to the incoming neutron beam. Elastically scattered neutrons were then counted while the temperature was continuously increased from 280 to 318 K at a rate of 0.16 K/min. The elastically scattered signals of the protein samples were then normalized by vanadium scattering and corrected for instrument effects, sample transmission and empty-cell scattering.

The instrumental energy resolution of 0.9 μeV (full width at half-maximum) allowed motions faster than about 1 ns to be probed. The instrument used neutrons with a wavelength of 6.27 Å and scattering vector modulus *Q* in the range of 0.2–1.9 Å^−1^. The observed scattering (Fig. 4) is closely similar for the two samples, indicating essentially identical dynamics in the nanosecond timescale. Previous extensive neutron scattering studies of PM dynamics under different conditions (for example)^36^,^38^ established that the low *Q* range (0.18 Å^−2^ < *Q*^2^ < 1.33 Å^−2^ in Fig. 4) is dominated by incoherent scattering that is well fitted by a Gaussian approximation. The deviation from the straight line and rise in scattering at higher *Q* values is due to a combination of the breakdown of the Gaussian approximation for the incoherent scattering and coherent scattering from lipid chains in the membrane. The intensity data in Fig. 4 are, therefore, fitted in the Gaussian approximation in the range 0.18 Å^−2^ < *Q*^2^ < 1.33 Å^−2^, according to equation (9)^28^.


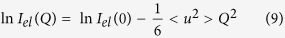


where the natural log of the scattered elastic intensity is plotted as a function of scattering vector squared to yield the MSD, <*u*^2^>. The mean effective force constant, <*k*′>. for the motions is obtained from the temperature dependence of the MSD (equation 10)^46^


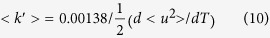


Corresponding MSD and <*k*′> for the two samples are in Fig. 5.

### Neutron liquids diffraction from aqueous solutions of ectoine

Two samples were examined on the D4C diffractometer at ILL (http://www.ill.eu/d4)^63^: solutions of 1.5 M of ectoine in water containing normal ectoine and heavy water (sample H-ectoine/D_2_O) and partially deuterated ectoine and heavy water (sample D-ectoine/D_2_O). The structure of water around ectoine was investigated through radial distribution functions g(r) obtained by Fourier transformations of the experimental structure factors S(*Q*) after the appropriate corrections (http://doi.ill.fr/10.5291/ILL-DATA.6-02-513). A wavelength of 0.499 Å was used with the incident beam perpendicular to the sample surface. The beam size was 44 mm (vertical) and 12 mm (horizontal). For these experimental conditions, the accessible q range was 0.22 Å^−1^ < q < 23.5 Å^−1^. A vanadium cylinder sample holder of 6 mm diameter was used. Standard corrections to account for differences in the relative efficiency of the microstrip multidetectors were applied. Measurements of the scattering corresponding to the empty bell jar and the sample holder were also carried out in order to take into account their contribution in the sample’s diffractograms. The corrections coming from the experimental conditions (absorption coefficients and multiple scattering contributions occurring either in the sample or in the container) were performed by the CORRECT code^64^. The background due to inelastic scattering was subtracted by using an empirical fitting, as described in reference^65^ for light atoms. Corrections for instrumental resolution and normalization to absolute units were performed by measuring a standard vanadium sample (considered as a fully incoherent scatterer).

## Additional Information

**How to cite this article**: Zaccai, G. *et al*. Neutrons describe ectoine effects on water H-bonding and hydration around a soluble protein and a cell membrane. *Sci. Rep.* **6**, 31434; doi: 10.1038/srep31434 (2016).

## Supplementary Material

Supplementary Information

## Figures and Tables

**Table 1:** Guinier parameters for the different samples and solvent conditions.

Sample	Solvent SLD (10^10^ cm^−2^)	*I*(0)/*N* (10^−16^ cm^2^)	*R*_g_ (Å)
H-MBP 2 M ect H_2_O	−0.074	0.142 ± 0.004	22.8 ± 0.7
H-MBP 3 M ect H_2_O	0.205	0.086 ± 0.003	24.1 ± 1.4
H-MBP 2 M ect D_2_O	5.70	0.154 ± 0.002	22.2 ± 0.25
H-MBP 3 M ect D_2_O	5.36	0.136 ± 0.002	23.6 ± 0.6
D-MBP 2 M ect H_2_O	−0.074	1.075 ± 0.015	25.6 ± 0.3
D-MBP 3 M ect H_2_O	0.205	1.025 ± 0.02	25.8 ± 0.4
D-MBP 2 M ect D_2_O	5.70	0.043 ± 0.003	30.7 ± 1.7
D-MBP 3 M ect D_2_O	5.36	0.086 ± 0.003	28.5 ± 0.7

**Table 2:** Measured H-bond parameters for ectoine solutions compared to average parameters for bulk D_2_O from Modig *et al*.^49^.

Parameter	Bulk D_2_O	Ectoine solution	Relative Difference
Intra-molecular			
O-D	0.97 Å	0.98 Å	<1%
D-D	1.53 Å	1.51 Å	<1%
D-O-D angle (α)	106°	100.4°	6%
Inter-molecular			
O-O	2.82 Å	2.80 Å	<1%
O-D	1.88 Å	1.93	3%
H-bond angle (β)	12°	22.36°	45%
