# Supplementary material for: Neutrons describe ectoine effects on water H-bonding and hydration around a soluble protein and a cell membrane
Source: Sci Rep. 2016 Aug 16;6:31434. doi: 10.1038/srep31434 (PMC4985633; doi:10.1038/srep31434)

Neutrons describe ectoine effects on water H-bonding and hydration around a soluble protein and a cell membrane

Giuseppe Zaccai[1,3,4,5*], Irina Bagyan[6], Jérôme Combet[1,7], Gabriel J. Cuello[1], Bruno Demé[1], Yann Fichou[3,4,5], François-Xavier Gallat[3,4,5], Victor M. Galvan Josa[1,8], Susanne von Gronau[2], Michael Haertlein[1], Anne Martel[1], Martine Moulin[1], Markus Neumann[6], Martin Weik[3,4,5], Dieter Oesterhelt[2*]

Author affiliations

(1) Institut Laue Langevin, F-38042 Grenoble, France; (2) Max-Planck-Institute of Biochemistry, Department of Membrane Biochemistry, Martinsried, Germany; (3) CNRS, IBS, F-38044 Grenoble, France; (4) CEA, IBS, F-38044 Grenoble, France; (5) Univ. Grenoble Alpes, IBS, F-38044 Grenoble, France; (6) Bitop, Stockumer Str. 28, 58453 Witten, Germany; (7) Institut Charles Sadron CNRS–UdS, 23 rue du Loess, BP 84047, 67034 Strasbourg Cedex 2, France; (8) Present address Galvan Josa: FaMAF, Córdoba, Argentina.

*Corresponding author: zaccai@ill.fr

Supplementary Information

In order to explore the effect of molar concentrations of ectoine on water structure, aqueous solutions of 1.5M ectoine were examined on the D4 liquids diffractometer. The experimental structure factors S(Q), obtained after subtraction of the incoherent background, of the following samples are displayed in Figure S1.

Figure S1. Structure factors S(Q) of 1.5M aqueous solutions of ectoine. H-ectoine/D_2O: Natural abundance ectoine in D_2O; D-ectoine/D_2O: D-labeled ectoine in D_2O; H-ectoine/H_2O: Natural abundance ectoine in H_2O.

The oscillatory scatter, beyond about $Q = 7$ Å^{-1}, in the H-ectoine/H_2O sample data reflects errors due to the small coherent signal / incoherent background ratio.

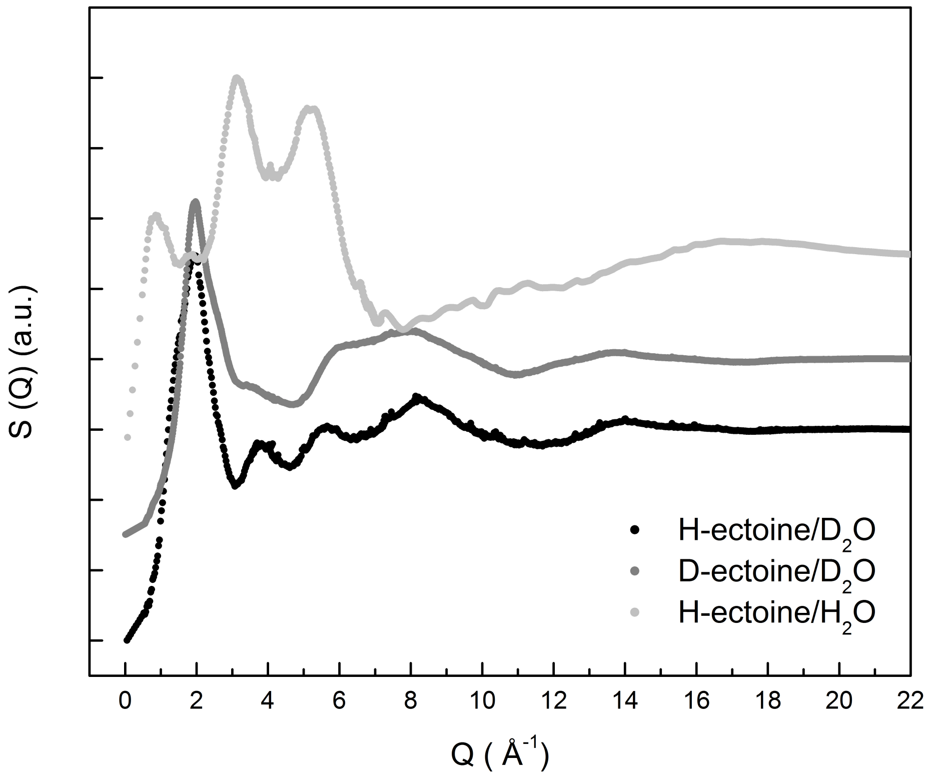

Supplement: Supplementary Information [file srep31434-s1.pdf]
